# Perioperative transfusion and the prognosis of colorectal cancer surgery: a systematic review and meta-analysis

**DOI:** 10.1186/s12957-018-1551-y

**Published:** 2019-01-05

**Authors:** Qian-Yun Pang, Ran An, Hong-Liang Liu

**Affiliations:** 1 Chongqing University Cancer Hospital and Chongqing Cancer Institute and Chongqing Cancer Hospital, Chongqing, China; 2 Department of Anesthesiology, Chongqing University Cancer Hospital and Chongqing Cancer Institute and Chongqing Cancer Hospital, NO.181, Hanyu Road, Shapingba district, Chongqing, 400030 China

**Keywords:** Transfusion, Colorectal cancer, Prognosis, Meta-analysis

## Abstract

**Background:**

Perioperative transfusion can reduce the survival rate in colorectal cancer patients. The effects of transfusion on the short- and long-term prognoses are becoming intriguing.

**Objective:**

This systematic review and meta-analysis aimed to define the effects of perioperative transfusion on the short- and long-term prognoses of colorectal cancer surgery.

**Results:**

Thirty-six clinical observational studies, with a total of 174,036 patients, were included. Perioperative transfusion decreased overall survival (OS) (hazard ratio (HR), 0.33; 95% confidence interval (CI), 0.24 to 0.41; *P* < 0.0001) and cancer-specific survival (CSS) (HR, 0.34; 95% CI, 0.21 to 0.47; *P* < 0.0001), but had no effect on disease-free survival (DFS) (HR, 0.17; 95% CI, − 0.12 to 0.47; *P* = 0.248). Transfusion could increase postoperative infectious complications (RR, 1.89, 95% CI, 1.56 to 2.28; *P* < 0.0001), pulmonary complications (RR, 2.01; 95% CI, 1.54 to 2.63; *P* < 0.0001), cardiac complications (RR, 2.20; 95% CI, 1.75 to 2.76; *P* < 0.0001), anastomotic complications (RR, 1.51; 95% CI, 1.29 to 1.79; *P* < 0.0001), reoperation(RR, 2.88; 95% CI, 2.05 to 4.05; *P* < 0.0001), and general complications (RR, 1.86; 95% CI, 1.66 to 2.07; *P* < 0.0001).

**Conclusion:**

Perioperative transfusion causes a dramatically negative effect on long-term prognosis and increases short-term complications after colorectal cancer surgery.

## Introduction

Patients with colorectal cancer often have accompanying anaemia or perioperative bleeding. Allogeneic transfusion becomes necessary in these cases. Some studies have found that perioperative transfusion could suppress the immune function and increase the recurrence and metastasis [1, 2], but others have not [3, 4]. One recent meta-analysis showed that perioperative transfusion could decrease the survival rate and increase the incidence rates of cancer recurrence and metastasis in colorectal cancer patients [5], but in that meta-analysis, low-quality studies were included, odds ratios (ORs) were used to extract the survival data, which was not appropriate, and the effects of censored data on the results were ignored. Until now, the effects of perioperative transfusion on the short- and long-term prognoses of the patients undergoing surgery for colorectal cancer are becoming increasingly intriguing. The effects of the volume and trigger of transfusion on the prognosis are unclear. Therefore, we conducted a systematic review and meta-analysis to address these issues and attempted to define the relationships between perioperative transfusion and short- or long-term prognosis in patients undergoing colorectal cancer surgery.

## Methods

We conducted our systematic review and meta-analysis in accordance with the methods recommended by the Preferred Reporting Items for Systematic Reviews and Meta-analysis (PRISMA) guidelines. There was no registered protocol.

### Literature search

The PubMed, Cochrane library, and Embase databases (from January 1990 to June 2018) were searched. The reference lists of the research studies and previous meta-analysis articles were also checked to find any further eligible trials.

The key words for the electronic search strategy included intestinal, intestine, bowel, colonic, colon, rectal, rectum, colorectal, cancer, tumour, carcinoma, neoplasm, transfusion, and blood transfusion. The citations to be searched were restricted to clinical studies and were published in English, the participants of our study were patients undergoing surgery for colorectal cancer, and the intervening measure was perioperative allogeneic transfusion. The exclusion criteria were comparison between allogeneic and autogenous transfusion or comparison between autogenous transfusion and no transfusion.

### Outcome parameters and data collection

The primary outcome of interest was overall survival (OS), while the secondary outcomes included disease-free survival (DFS), cancer-specific survival (CSS), and postoperative complications. OS was defined as the time from surgery to death from any cause. DFS was defined as the time from surgery to a recurrence or death from any cause. CSS was defined as the time from surgery to death from cancer recurrence or metastasis. Data were extracted and collected by two authors independently, and disagreements were resolved by discussion and consensus among all authors.

### Quality assessment

The quality of publications was judged by the Newcastle-Ottawa Scale (NOS); a quality review of the data obtained from each study was performed on the basis of case selection, comparability, and outcome reporting. The highest score was 9 stars; a study with an NOS score greater than or equal to 7 stars was defined as a high-quality study, and if the NOS score was less than 7 stars, the study was excluded.

### Statistical analysis

Meta-analysis was performed using Stata 12.0 software (StataCorp LP, US). The hazard ratios (HRs) with 95% confidence intervals (CIs) were calculated for OS, DFS, and CSS, and the risk ratios (RRs) with 95% CIs were calculated for postoperative complications. The HRs were extracted from the multivariable analysis when both univariable and multivariable analyses were available, and Engauge Digitizer 4.1 and Adobe Photoshop software were used for the extraction of HR [6]. Statistical heterogeneity was assessed using the chi-square test and *I*^*2*^ statistics; *I*^2^ ≥ 50% (*P* ≤ 0.1) indicated significant heterogeneity, and the random-effects model was used, and the fixed-effects model was used when *I*^2^ < 50% (*P* > 0.1) [7]. Subgroup and sensitivity analyses were performed to explore the source and size of heterogeneity among studies when necessary. Publication bias was evaluated by the Egger test, and *P* ≥ 0.05 represented no statistical significance in publication bias.

## Results

### Literature search and characteristics of eligible trials

**Fig. 1:**
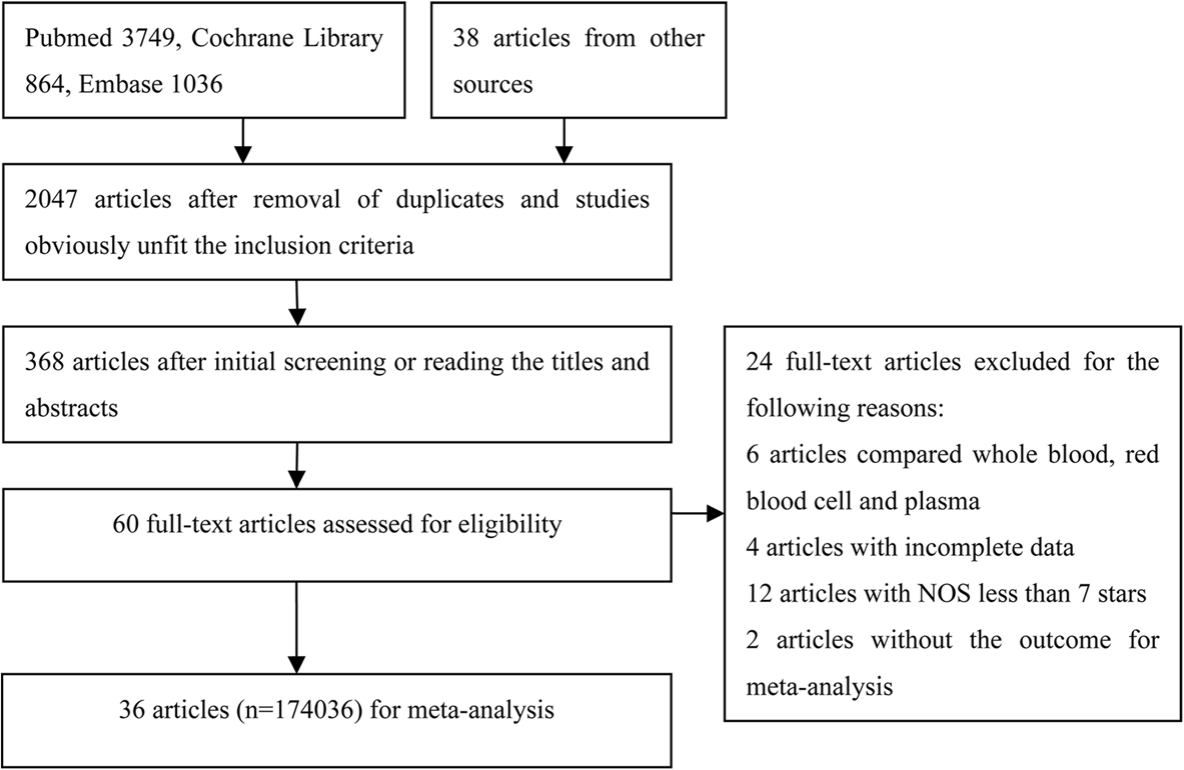
Flow chart of the study screening for this meta-analysis

We identified 5687 potential articles: 3749 articles from PubMed, 864 articles from Cochrane library, 1036 articles from Embase, and 38 articles from other sources. Sixty relevant articles were left after initial screening and reading the titles or abstracts, and 36 clinical observational studies with a total of 174,036 patients were ultimately included [1–4, 8–39]. The details of the screening process are presented in Fig. 1. These 36 studies were conducted in different countries and were published from 1990 to 2018. The characteristics and the qualities of these studies are presented in Table 1.

**Table 1:** Characteristics of the trials

ID	Country	Tumour type	Sample size			Transfusion trigger	Type of blood products	Outcomes	NOS (stars)
			BT+	BT−	Total				
1 Tarantino I 2013 [1]	Switzerland	Colonic	148	161	309	–	–	OS	7
2 Gunka I 2013 [2]	Czech	Colorectal	132	451	583	–	–	OS	7
3 Amri R 2017 [3]	USA	Colonic	305	1118	1423	–	–	OS, CSS	8
4 Morner MEM 2016 [4]	Sweden	Colorectal	199	297	496	–	–	OS	8
5 Qiu L 2015 [8]	China	Colorectal	803	601	1404	Hb < 6 g/dL, Hb 6–10 g/dL according to cardiopulmonary function	–	OS, postoperative complications	7
6 Miki C 2006 [9]	Japan	Colorectal	35	82	117	–	–	OS, postoperative complications	8
7 Meng J 2013 [10]	China	Colonic	259	211	470	Hb < 8 g/dL	–	OS, postoperative complications	7
8 Talukder Y 2014 [11]	Australia	Colorectal	423	947	1370	–	–	OS, DFS, CSS, postoperative complications	8
9 Due SL 2012 [12]	Australia	Colorectal	151	654	805	–	–	CSS, postoperative complications	7
10 Jagoditsch M 2006 [13]	Australia	Rectal	471	126	597	Preoperative Hb < 8 g/dL, intraoperative bleeding > 500 mL or Hb < 10 g/dL	–	OS, DFS, postoperative complications	7
11 Lobaziewicz W 2008 [14]	Poland	Colorectal	122	135	257	Intraoperative bleeding > 1000 mL or Hct < 30%	RBC	OS, CSS, DFS	8
12 Kaneko M 2015 [15]	Japan	Colorectal	23	85	108	–	Allogeneic red blood cell	OS	8
13 Nursal TZ 2006 [16]	Turkey	Colorectal	61	272	333	–	Whole blood or RBC	OS	7
14 Li XX 2015 [17]	China	Colonic	614	461	1075	Hb < 6 g/dL, Hb 6–10 g/dL according to cardiopulmonary function	–	OS, postoperative complications	7
15 Halabi WJ 2013 [18]	USA	Colorectal	3815	23,305	27,120	–	RBC	OS, postoperative complications	8
16 Warschkow R 2014 [19]	Switzerland	Rectal	217	184	401	–	Prestored allogeneic blood	OS, DFS	7
17 Koch M 2011 [20]	Germany	Colorectal	135	396	531	Hb 8–10 g/dL according to cardiopulmonary function	–	Postoperative complications	7
18 Ghinea R 2013 [21]	Italy	Colorectal	68	133	201	–	–	OS, DFS, postoperative complications	7
19 Skanberg J 2007 [22]	Sweden	Colorectal	298	344	642	–	LDB or RBC	OS	8
20 Mynster T 2000 [23]	Denmark	Colorectal	288	452	770	–	SAGM and/or FFP	Postoperative complications	8
21 Patel SV 2017 [24]	Canada	Colonic	2009	5189	7198	–	–	OS, CSS	9
22 van de Watering LMG2001 [25]	Netherlands	Colorectal	446	251	697	–	LDB or RBC	OS	7
23 Papageorge CM 2016 [26]	England	Colorectal	2073	58,712	72,011	–	Whole blood or RBC	Postoperative complications	7
24 Benoist S 2001 [27]	France	Rectal	72	140	212	Hb < 8 g/dL	–	Postoperative complications	7
25 Jensen LS 2005 [28]	Denmark	Colorectal	249	320	569	–	LDB or RBC	OS, postoperative complications	9
26 Mynster T 2001 [29]	Denmark	Colorectal	452	288	740	–	SAGM and/or FFP	OS	7
27 Aquina CT 2016 [30]	USA	Colorectal	6927	17,303	24,230	–	–	OS, CSS, postoperative complications	8
28 Mazzeffi M 2017 [31]	USA	Colonic	1845	23,388	24,733	–	RBC	Postoperative complications	8
29 Van Osdol AD 2015 [32]	USA	Colorectal	110	365	475	Postoperative Hb < 7 g/dL, preoperative Hb < 8.4 g/dL	–	OS, DFS, postoperative complications	7
30 Sánchez-Velázquez P 2018 [33]	Spain	Colonic			363	–	–	DFS, CSS	8
31 Molland G 1995 [34]	Australia	Colorectal	223	210	433	–	All kinds of blood products	OS	8
32 Cheslyn-Curtis S 1990 [35]	UK	Colorectal	591	370	961	–	–	OS	7
33 Donohue JH 1995 [36]	USA	Colorectal	446	605	1051	–	Whole blood or RBC or plasma	OS	8
34 Tartter PI 1992 [37]	USA	Colorectal	110	229	329	–	RBC	DFS	7
35 Garau I 1994 [38]	Spain	Colorectal	348	338	686	–	Whole blood or RBC or plasma	OS	7
36 Edna TH 1994 [39]	Norway	Colorectal	236	100	336	Hb < 9 g/dL or bleeding > 20% blood volume	SAGM	OS	8

*LDB* leucocyte-depleted blood products, *RBC* packed red blood cells, *SAGM* buffy coat-depleted red cells suspended in saline, adenine, glucose, and mannitol, *FFP* fresh-frozen plasma

#### Results of meta-analysis

##### Overall survival (OS)

**Fig. 2:**
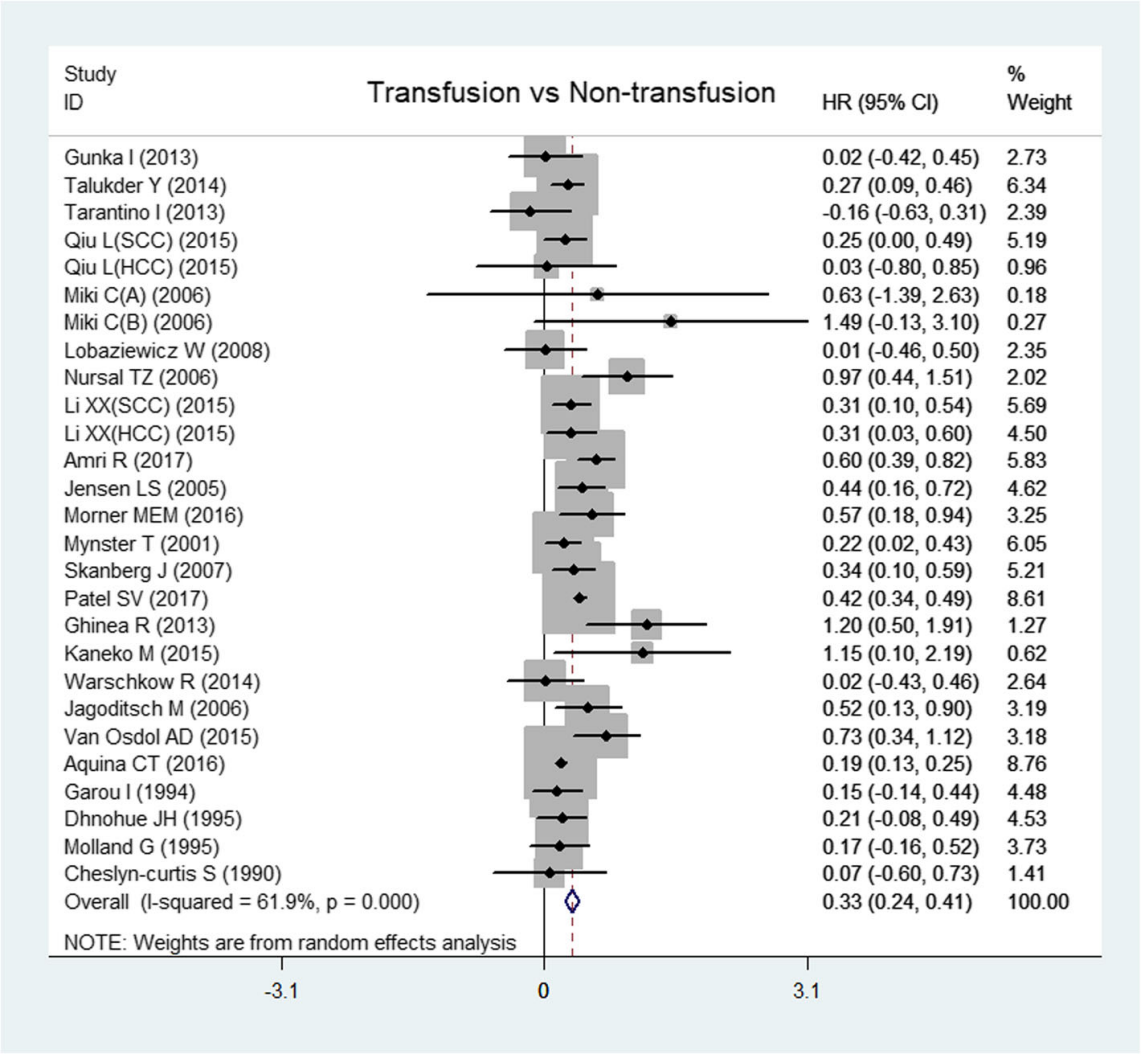
Forest plot of overall survival after perioperative transfusion. SCC indicates sporadic colorectal cancer, HCC indicates hereditary colorectal cancer, group A in Miki C 2006 indicates the patient who received transfusion because of preoperative anaemia, and group B in Miki C 2006 indicates the patient who received transfusion because of excessive operative blood loss

Data on OS were from 24 articles [1–4, 8, 9, 11, 13, 14, 16, 17, 19, 21, 22, 24, 28–30, 32–36, 38]. The random-effects model showed that transfusion could decrease OS significantly (HR, 0.33; 95% CI, 0.24 to 0.41; *I*^2^ = 61.9%; *P* < 0.0001; Fig. 2). There was no significant publication bias from the Egger test (*P* = 0.297).

**Fig. 3:**
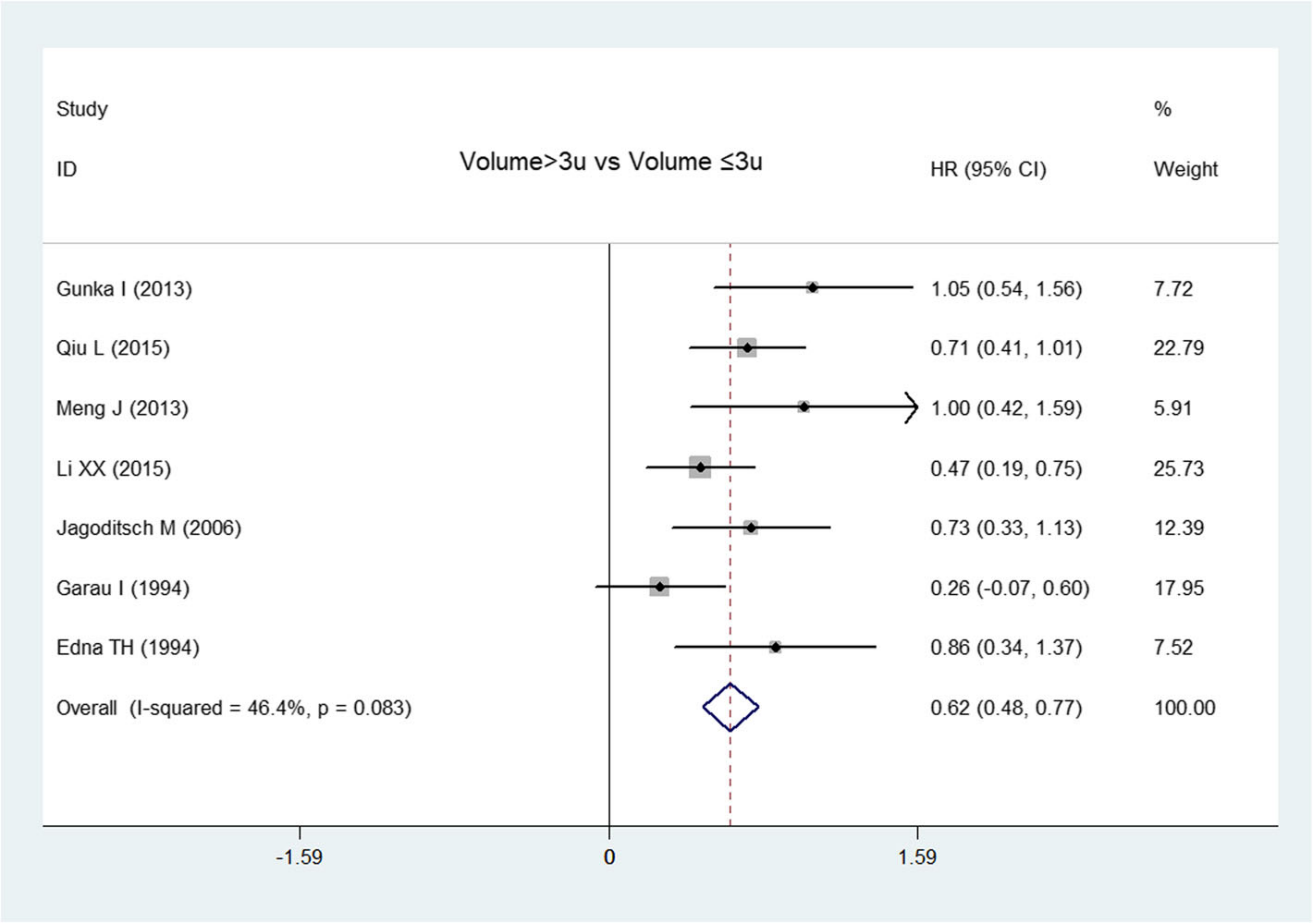
Forest plot of the effect of transfusion volume on overall survival

Seven articles [2, 8, 10, 13, 17, 38, 39] reported the influence of transfusion volume (> 3 u and ≤ 3 u) on OS. The fixed-effects model showed that OS was lower in the large transfusion volume group (> 3 u) compared with those in the small transfusion volume group (≤ 3 u) (*I*^2^ = 46.4%, HR = 0.62, 95% CI 0.48–0.77, *P* < 0.0001) (Fig. 3). There was no significant publication bias from the Egger test (*P* = 0.072).

**Fig. 4:**
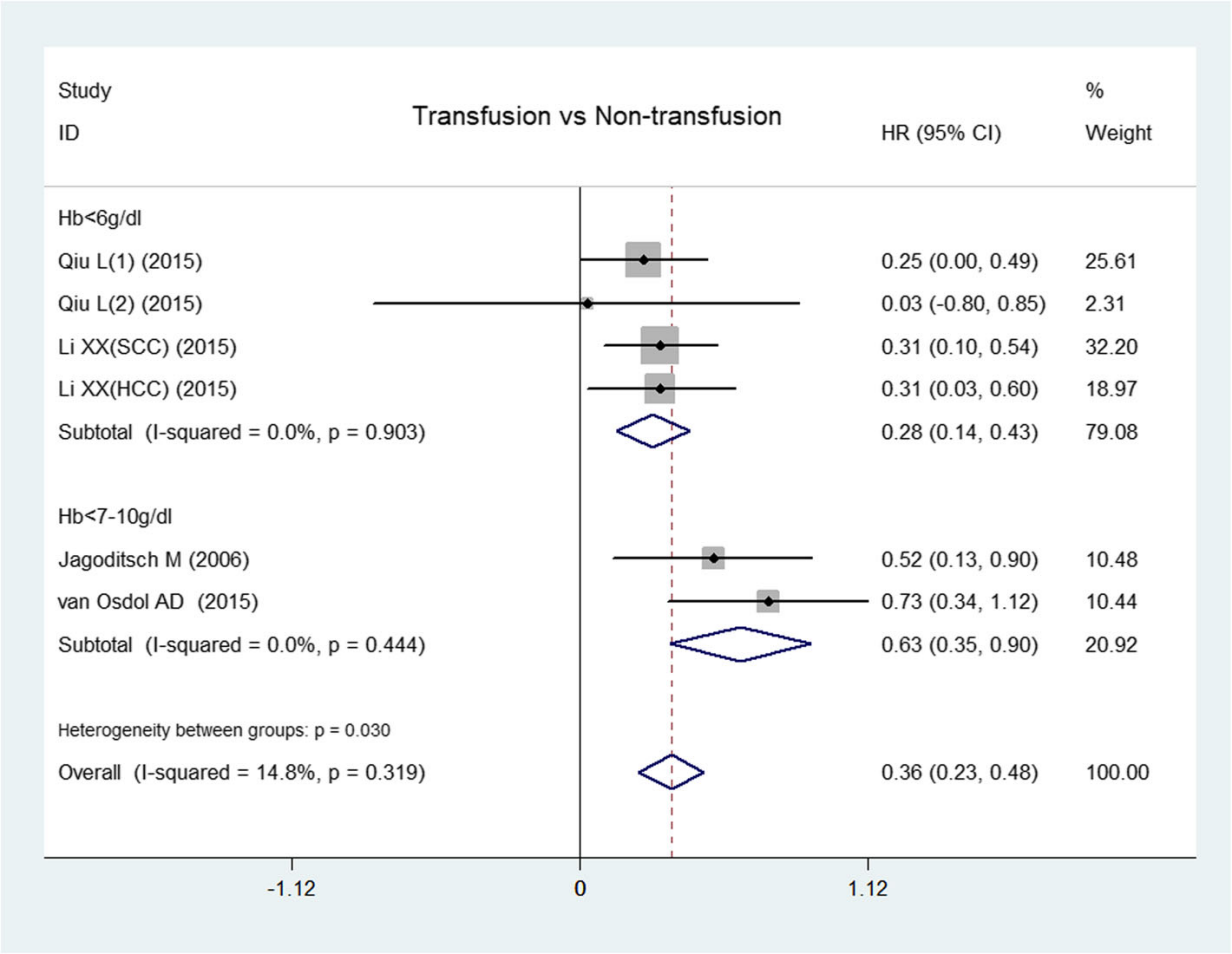
Forest plot of the effect of transfusion trigger on overall survival

Nine articles reported the trigger of transfusion. The comparison between transfusion and non-transfusion on OS was from five of these articles [8, 13, 14, 17, 32]; in the five articles, one [14] used intraoperative bleeding > 1000 ml as a trigger of transfusion, and the other four articles were included in the following meta-analysis. The triggers were Hb ≤ 6 g/dl and Hb ≤ 7–10 g/dl, and the results showed that transfusion could reduce OS compared with non-transfusion if the trigger level was either Hb ≤ 6 g/dl (*I*^2^ = 0%, HR = 0.28, 95% CI 0.14–0.43, *P* < 0.0001) or Hb ≤ 7–10 g/dl (*I*^2^ = 0%, HR = 0.63, 95% CI 0.35–0.90, *P* < 0.0001) (Fig. 4). There was no significant publication bias from the Egger test (*P* = 0.667).

##### Disease-free survival (DFS) and cancer-specific survival (CSS)

**Fig. 5:**
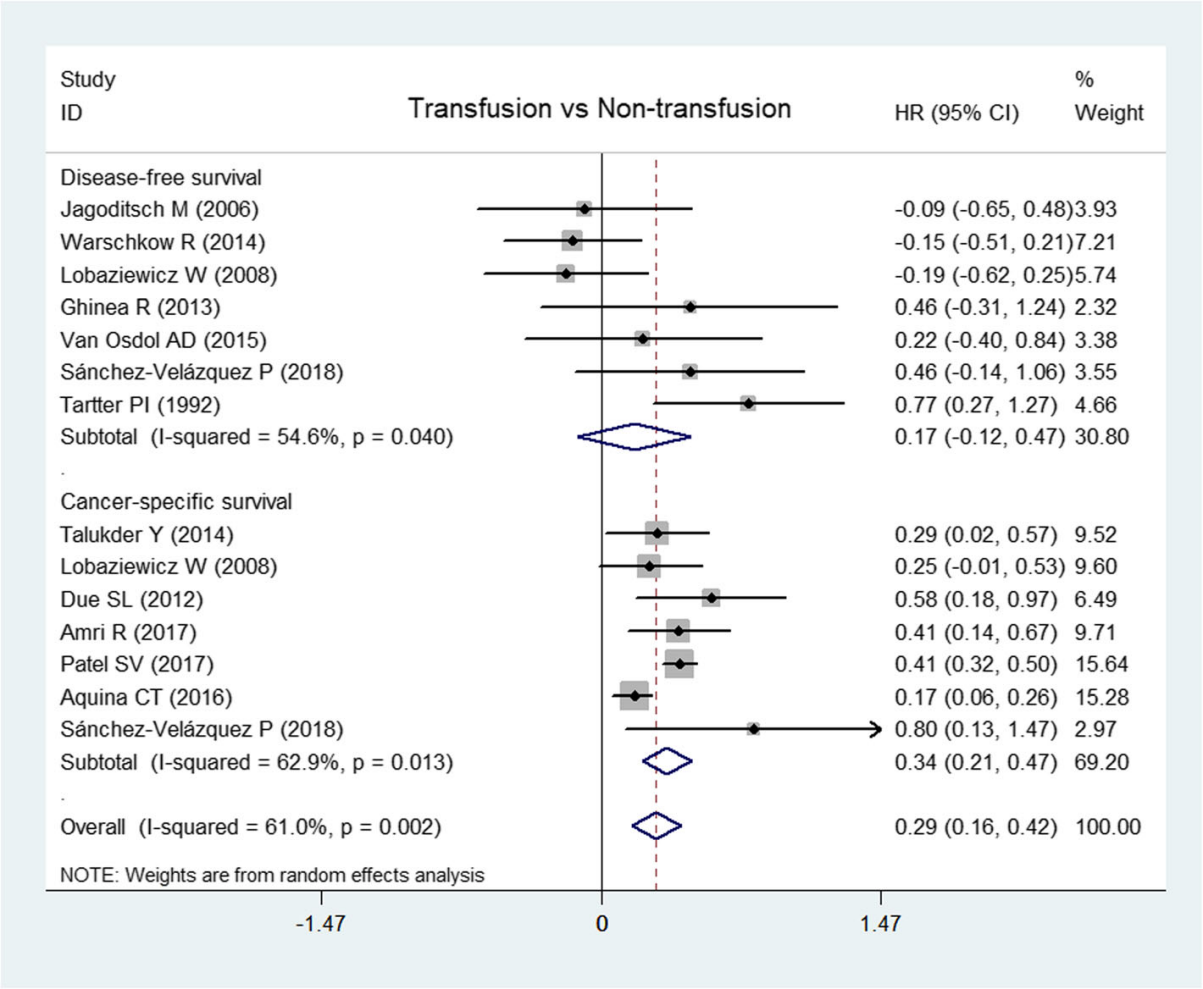
Forest plots of disease-free survival and cancer-specific survival after perioperative transfusion

Data on *DFS and CSS* were from 7 [13, 14, 19, 21, 32, 33, 37] and 7 articles [3, 11, 12, 14, 24, 30, 33], respectively. The random-effects model showed that transfusion could decrease CSS significantly (HR, 0.34, 95% CI, 0.21 to 0.47, *I*^2^ = 62.9%; *P* < 0.0001) but did not affect DFS (HR, 0.17; 95% CI, − 0.12 to 0.47; *I*^2^ = 54.6%; *P* = 0.248) (Fig. 5). There was no significant publication bias from the Egger test (*P* = 0.912).

##### Postoperative complications

**Fig. 6:**
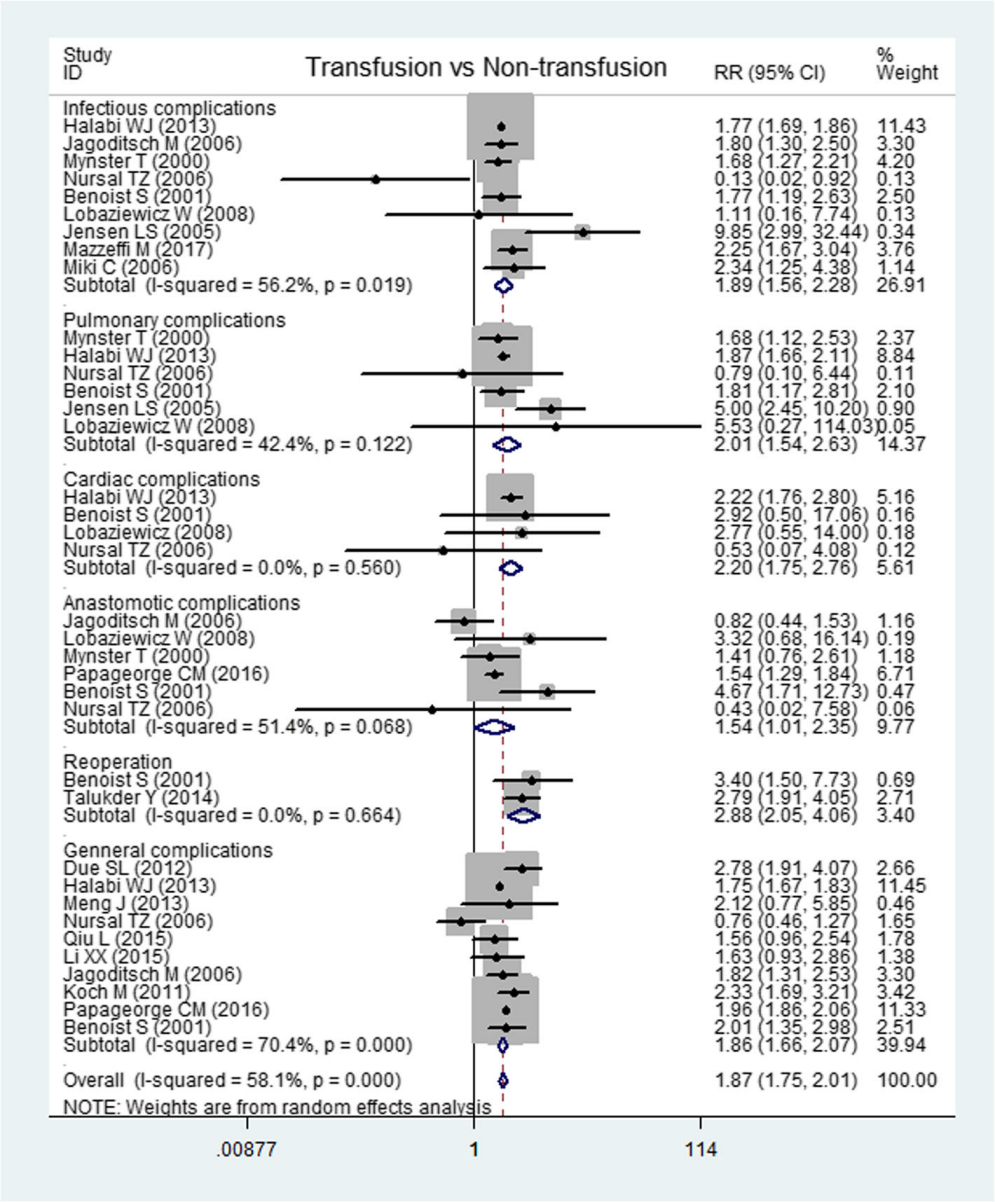
Forest plots of postoperative complications after perioperative transfusion

Data on 30- or 60-day postoperative infectious complications (wound and urinary infections), pulmonary complications (pneumonia, respiratory failure, and pulmonary embolism), cardiac complications (myocardial infarction, angina, cardiac arrest, and arrhythmia), anastomotic complications (anastomotic fistula and bleeding), reoperation, and general complications were from 9 [9, 13, 14, 16, 18, 23, 27, 28, 31], 6 [14, 16, 18, 23, 27, 28], 4 [14, 16, 18, 27], 6 [13, 14, 16, 23, 26, 27], 2 [11, 27], and 10 [8, 10, 12, 13, 16–18, 20, 26, 27] articles respectively. The meta-analysis showed that transfusion could increase infectious complications (RR, 1.89, 95% CI, 1.56 to 2.28; *I*^2^ = 56.2%; *P* < 0.0001), pulmonary complications (RR, 2.01; 95% CI, 1.54 to 2.63; *I*^2^ = 42.4%; *P* < 0.0001), cardiac complications (RR, 2.20; 95% CI, 1.75 to 2.76; *I*^2^ = 0%; *P* < 0.0001), anastomotic complications (RR, 1.51; 95% CI, 1.29 to 1.79; *I*^2^ = 51.4%; *P* < 0.0001), reoperation (RR, 2.88; 95% CI, 2.05 to 4.05; *I*^2^ = 0%; *P* < 0.0001), and general complications (RR, 1.86; 95% CI, 1.66 to 2.07; *I*^2^ = 70.4%; *P* < 0.0001) (Fig. 6), and there was no significant publication bias from the Egger test (*P* = 0.541).

##### Subgroup and sensitivity analyses for OS

Table 2 shows the subgroup analysis for OS. Publication dates, sample size, and study region did not influence the effects. However, data on OS in rectal cancer patients were only from two articles, and the subgroup analysis showed that transfusion had no significant effect on OS in rectal cancer patients, which was different from the finding for colorectal cancer or colon cancer patients. The sensitivity analysis showed that the results of the effect of transfusion on OS were not changed when any suspicious study was omitted.

**Table 2:** Subgroup analyses for overall survival

Stratified	No. of studies	*I*^2^ (%)	HR (95% CI)	*P* value
Publication date (year)				
1990–2000	4	0	0.17 (0, 0.34)	0.049
2001–2010	7	4.5	0.32 (0.19, 0.45)	< 0.0001
2011–2018	14	74.7	0.37 (0.25, 0.48)	< 0.0001
Study size (no. of patients)				
< 500	9	63.1	0.42 (0.12, 0.71)	0.005
≥ 500	5	63.1	0.32 (0.23, 0.40)	< 0.0001
Region				
Asia	4	0	0.31 (0.17, 0.45)	< 0.0001
Europe	12	56.9	0.29 (0.12, 0.46)	0.0001
America	5	88.7	0.40 (0.22, 0.57)	< 0.0001
Australia	3	0	0.29 (0.14, 0.44)	< 0.0001
Surgical type				
Colorectal	18	48.6	0.31 (0.21, 0.41)	< 0.0001
Colonic	4	59.8	0.37 (0.23, 0.52)	< 0.0001
Rectal	2	63.8	0.28(− 0.21, − 0.77)	0.258

*No.* number, *HR* hazard ratio, *95% CI* 95% confidence interval

## Discussion

Colorectal cancer is the most common human cancer. In the past few decades, many retrospective studies have focused on the effects of perioperative transfusion on short- and long-term prognoses in colorectal cancer patients, and a larger transfusion volume seems to have a poorer prognosis. It is very important to evaluate the trigger of transfusion and the influence of the volume of transfusion on prognosis to optimise perioperative transfusion and improve the outcome in colorectal cancer patients. Our meta-analysis reviewed the current available literature on perioperative transfusion and the prognosis of colorectal cancer surgery and extracted the survival data by HR method, which is more precise than the OR or RR methods.

Our results showed that perioperative transfusion could reduce OS and CSS and could increase the incidence of postoperative complications. Inflammatory and immunosuppressive mediators were proved to be associated with the development of recurrence and metastasis [40, 41], and transfusion could accelerate tumour progression by inducing an inflammatory response and immunosuppression [42]. In our meta-analysis, transfusion could reduce OS and CSS but had no effect on DFS. The possible reasons for this effect are that surgery, anaesthesia-related factors, and cancer staging can affect DFS, in addition to transfusion.

Allogeneic blood products release inflammatory factors during storage and can cause immunosuppression, including inhibiting NK cell activity and decreasing the Th1/Th2 ratio; eventually, infectious complications are increased after transfusion [42–46]. It has been reported that postoperative intra-abdominal infection is an independent prognostic factor of DFS and disease-specific survival in patients with colon cancer [33]. In our meta-analysis, transfusion also increased the incidence of cardiopulmonary complications, anastomotic complications, and reoperation, which suggested that postoperative complications might have a negative impact on oncologic outcome. There are only two articles (1582 patients) included that addressed the incidence of reoperation; thus, more studies are needed to confirm the result.

Our meta-analysis showed that the poor overall survival was closely related with the transfusion volume. Large amounts of blood products can generate more active biochemical substances, including vascular endothelial growth factors and plasminogen activator inhibitors, and are more likely to promote the tumour angiogenesis and tumour cell proliferation and migration [47]; together with surgical stress, large-volume transfusions may cause more immunosuppression [18]. One study showed that restrictive transfusion (transfusion trigger: Hb < 8 g/dl) could not improve the survival rate, especially in a high-risk group of elderly patients with cardiovascular disease [48], and our meta-analysis showed that restrictive (transfusion trigger: Hb ≤ 6 g/dl) or liberal transfusion (transfusion trigger: Hb ≤ 7–10 g/dl) could decrease the OS significantly compared with non-transfusion. However, until now, there have been no direct comparisons between different transfusion triggers on prognosis, and very few articles report the trigger of transfusion. Since it is relatively rare in clinical circumstances that bleeding over 1000 ml as a transfusion trigger, we performed a sensitivity analysis, and the results showed that the effect of transfusion on OS was not changed when the article was omitted. Anaemia itself could negatively affect the prognosis of malignancy and could increase the risk for overall mortality, and the presence of anaemia was an independent risk factor for postoperative complications and a longer hospital stay after colon surgery [49]; therefore, preoperative therapy for anaemia was recommend to reduce the need for blood transfusions, and iron supplements have no influence on tumour progression [50].

It is known that allogeneic transfusion can aggravate perioperative immunosuppression in cancer patients, and autogenous transfusion seems to be superior to allogeneic transfusion [51, 52], but Harlaar et al. did not find any benefit from autologous transfusion compared with allogeneic transfusion after long-term follow-up in colorectal cancer patients [53]. The clinical data for autogenous transfusion in cancer patients are sparse, and the safety of autogenous transfusion is still a big concern in the clinic, as autogenous transfusion has the potential risk to induce iatrogenic metastasis.

There are some limitations of our meta-analysis. All of the included articles were observational studies, published from 1990 to 2018. The methods and drugs for anaesthesia and analgesia were not mentioned in these trials and may be different to some extent. Some other risk factors such as preoperative Hb level, different kinds and storage durations of blood products, operation duration, and the staging of cancer might affect the prognosis of colorectal cancer surgery. According to the subgroup analysis, different types of surgical procedure had different outcomes. However, most of the included articles did not describe colonic and rectal cancers surgery separately, so that the articles including colon cancer or rectal cancer are taken together in our meta-analysis. Therefore, prospective controlled clinical trials with large sample sizes need to be conducted to verify the results of our meta-analysis.

## Conclusion

The results of our meta-analysis suggest that perioperative transfusion causes a dramatically negative effect on long-term prognosis and increases the short-term complications after colorectal cancer surgery.

## Abbreviations

CSS: Cancer-specific survival
DFS: Disease-free survival
OS: Overall survival
